# Umbilical Cord Blood as a Source of Less Differentiated T Cells to Produce CD123 CAR-T Cells

**DOI:** 10.3390/cancers14133168

**Published:** 2022-06-28

**Authors:** Blandine Caël, Jeanne Galaine, Isabelle Bardey, Chrystel Marton, Maxime Fredon, Sabeha Biichle, Margaux Poussard, Yann Godet, Fanny Angelot-Delettre, Christophe Barisien, Christophe Bésiers, Olivier Adotevi, Fabienne Pouthier, Francine Garnache-Ottou, Elodie Bôle-Richard

**Affiliations:** 1 RIGHT Interactions Greffon-Hôte-Tumeur/Ingénierie Cellulaire et Génique, EFS BFC, INSERM, Univ. Bourgogne Franche-Comté, F-25000 Besançon, France; blandine.cael@efs.sante.fr (B.C.); jeanne.galaine@efs.sante.fr (J.G.); chrystel.marton@chu-lille.fr (C.M.); maxime.fredon@efs.sante.fr (M.F.); sabeha.biichle@univ-fcomte.fr (S.B.); margaux.poussard@efs.sante.fr (M.P.); yann.godet@efs.sante.fr (Y.G.); fanny.delettre@efs.sante.fr (F.A.-D.); olivier.adotevi@univ-fcomte.fr (O.A.); francine.garnache@efs.sante.fr (F.G.-O.); 2 Activité d’Ingénierie Cellulaire et Tissulaire, Etablissement Français du Sang Bourgogne/Franche-Comté, F-25000 Besançon, France; isabelle.bardey@efs.sante.fr (I.B.); fabienne.pouthier@efs.sante.fr (F.P.); 3 Allogenic Stem Cell Transplantation Unit, Department of Hematology, CHU Lille, F-59000 Lille, France; 4 EFS Bourgogne/Franche-Comté, F-25000 Besançon, France; christophe.besiers@efs.sante.fr; 5 Département Collecte et Production de PSL, Etablissement Français du Sang Bourgogne Franche-Comté, F-25000 Besançon, France; christophe.barisien@efs.sante.fr; 6 Service Oncologie Médicale, CHU Besançon, F-25000 Besançon, France

**Keywords:** Umbilical Cord Blood, CAR-T cells, adoptive cell therapy, allogeneic immunotherapy, CD123

## Abstract

**Simple Summary:**

We used fresh or thawed Umbilical Cord Blood (UCB) to produce CAR-T cells directed against CD123, and we compared their functionality to Peripheral Blood (PB) CAR-T cells. T cells expressing CD123 CAR, derived from UCB, was exhibited through a high transduction rate, activation status, and cytotoxic potential in vitro as PB derived CAR-T cells. Moreover, we obtained T cells that had a less differentiated profile than the PB-derived T cells. UCB derived CAR-T can significantly control tumor progression in mice models. CAR-T obtained from thawed or fresh UCB gives the same results.

**Abstract:**

Chimeric Antigen Receptor (CAR) therapy has led to great successes in patients with leukemia and lymphoma. Umbilical Cord Blood (UCB), stored in UCB banks, is an attractive source of T cells for CAR-T production. We used a third generation CD123 CAR-T (CD28/4-1BB), which was previously developed using an adult’s Peripheral Blood (PB), to test the ability of obtaining CD123 CAR-T from fresh or cryopreserved UCB. We obtained a cell product with a high and stable transduction efficacy, and a poorly differentiated phenotype of CAR-T cells, while retaining high cytotoxic functions in vitro and in vivo. Moreover, CAR-T produced from cryopreserved UCB are as functional as CAR-T produced from fresh UCB. Overall, these data pave the way for the clinical development of UCB-derived CAR-T. UCB CAR-T could be transferred in an autologous manner (after an UCB transplant) to reduce post-transplant relapses, or in an allogeneic setting, thanks to fewer HLA restrictions which ease the requirements for a match between the donor and recipient.

## 1. Introduction

In recent years, immunotherapy has emerged as a new pillar of cancer treatment, especially as an adoptive cell therapy. Chimeric Antigen Receptor (CAR) T cell therapy enhances the potency of the anti-tumor T cell response by redirecting T cells to a tumor-associated antigen [1]. Thus, CAR-T immunotherapy is a breakthrough approach to cancer immunotherapy that has shown a substantial benefit in patients suffering from relapsed or refractory B cell malignancies [2]. Indeed, more than 800 CAR-T clinical trials have been initiated to date [3,4].

For now, all the CAR-T therapies that have been authorized by health authorities are autologous CAR-T therapies (i.e., produced from the patient’s T cells); however, the autologous strategy presents some drawbacks, especially in terms of the quality and quantity of T cells that are produced by the patient, which may be insufficient. Moreover, the very high cost of autologous therapies make it difficult to scale up production [5,6,7,8]. In order to overcome these challenges, many projects are focusing on the development of allogeneic CAR-T, an “off-the-shelf” product derived from banks of healthy donor T cells [9,10]. This approach would enable the injection of high-quality T cells into patients, in sufficient quantities, and in a shorter period of time. Although they are mainly developed from adult Peripheral Blood (PB) T cells, CAR-T cells could also be generated from Umbilical Cord Blood (UCB)-derived T cells. In fact, in 1991, UCB banks were created in the USA, and in 1995, they opened in France; currently, there are more than 805,000 units of cryopreserved UCB that are ready for use [11]. Today, UCB is only authorized for stem cell transplantation, and its use is decreasing each year. Thus, the development of novel cell therapy products from UCB could be a solution, as it might diversify the activities of UCB banks [12,13]. Moreover, UCB contains T cells with completely different immunological and phenotypic properties from PB [14,15]. More than 85% of UCB-derived T cells have a © phenotype, allowing them to induce fewer graft-versus-host-diseases (GVHDs) during UCB transplantation [16]. Moreover, they express significantly lower markers of exhaustion (PD1, LAG3, TIM3) in comparison with PB-derived T cells, allowing them to have long-term persistence and efficiency [17]. For these reasons, UCB-derived T cells have been garnering more interest [18,19]. The production of UCB-derived CAR-T is still in its infancy, but it presents many putative advantages.

Another issue that has emerged from the real-world (patients treated with CD19 CAR-T) is the importance of the in vivo persistence of CAR-T, which, in turn, is correlated with relapse and therapeutic efficacy [20,21,22]. CAR-T persistence depends on variable parameters, such as the CAR construction, costimulatory domain(s), level of CAR-T activation, the production strategy, and so on. The second and third generations of CAR-T are positively correlated to higher in vivo CAR-T persistence [23,24,25]. In third generation CAR-T cells, both 4-1BB and CD28 domains promote tonic signaling, and they enhance its in vivo persistence [25,26,27].

To improve CAR-T persistence, another strategy relies on injecting T cells with a poor differentiation profile. Naïve (T_N_), Stem Cell Memory (T_SCM_), and Central Memory (T_CM_) T cells have high capacities for survival and proliferation, which yield better in vivo persistence, and consequently, longer anti-tumor capacities than more differentiated T cells, such as effector memory (T_EM_) or effector (T_EFF_ or T_EMRA_) [28,29,30]. UCB, which is mostly composed of T_N_, would be an ideal source for the development of less differentiated CAR-T cells [16,31]. The use of cytokines, such as IL-7 and IL-15 during the culturing of CAR-T, also favor this phenotype, and they enhance CAR-T cell antitumor activity [32,33,34].

We previously demonstrated the anti-leukemia efficacy and safety of a third generation lentiviral, CD28/4-1BB CAR-T (CD123 CAR-T), which targeted CD123 and had high antitumor activity in different blastic plasmacytoid dendritic cell neoplasm (BPDCN) models [35]. Here, we tested the ability of fresh and cryopreserved UCB in terms of whether it could produce CD123 CAR-T in combination with IL-7 and IL-15 cytokines. We evaluated the functionality of UCB-derived CAR-T (UCB CAR-T) in comparison with PB-derived CAR-T.

## 2. Materials and Methods

### 2.1. T Cell Collection

Fresh Umbilical Cord Blood (UCB) units for research purposes were obtained from the French Blood Transfusion Center Cord Blood Bank (Cell Therapy Unit, EFS Besançon, Besançon, France). Peripheral Blood (PB) was collected from healthy adult donors using prepared apheresis kits. Written, informed consent was obtained from all donors.

### 2.2. T Cell Activation and Expansion

Umbilical Cord Blood Mononuclear Cell (UCBMC) and Peripheral Blood Mononuclear Cell (PBMC) isolation was performed by using the density-gradient technique with Ficoll-Hypaque (Eurobio, Les Ullis, France) within 24 h after the blood draw. T cell isolation and activation was performed by incubating the samples with aCD3/CD28 magnetic beads (4:1 bead-to-cell ratio, Gibco, Grand Island, NY, USA). T cells were expanded for 13 days in the RPMI 1640 medium (RPMI, Gibco Thermo Fisher Scientific, Waltham, MA, USA), which was supplemented with 10% heat-inactivated human serum (EFS local production), 1% Penicillin (100 IU/mL), and Streptomycin (1000 µg/mL) (PS, Eurobio, France), in the presence of optimized doses of IL-7 (IL-7, 20 IU/mL, Miltenyi Biotec, Bergisch Gladbach, Allemagne, Germany) and IL-15 (10 IU/mL, Miltenyi Biotec). Cells were counted every two days, and the RPMI 1640 medium with IL-7 and IL-15 was added every 2–3 days in order to maintain the T cell count between 0.5–1 × 10^6^ cells/mL. 

### 2.3. Cell Line and CAR Construction

The CD123^+^ BPDCN cell line, CAL-1 (provided by Dr. Maeda, Nagasaki University, Japan), was conserved in an internal master cell bank for non-ambiguous identification. Cell lines were cultured in the Roswell Park Memorial Institute 1640 medium, supplemented with 10% heat-inactivated, endotoxin-free, fetal calf serum (Invitrogen, Cergy-Pontoise, France), 1% Penicillin (100 IU/mL), and Streptomycin (1000 µg/mL).

The CD123 CAR lentivirus has been described in previous studies [35]. In sum, the CAR contains an extracellular single chain variable fragment (scFv) that is specific for CD123, and multiple costimulatory domains, including CD28, 4-1BB, and CD3z. The lentiviral construct encodes the third generation CAR and truncated protein CD19, which is a reporter gene, to monitor and select the transduced T cells.

### 2.4. CAR-T Cell Generation from Fresh UCB

Lentivirus encoding CD123-CAR was spin-transduced into activated CD3^+^ T cells on day 2 (800 g, 32 °C for 1 h to a MOI of 5–10). Thereafter, T cells were cultured in the RPMI 1640 medium containing 10% heat-inactivated human serum and 1% PS in a 37 °C, 5% CO_2_ humidified incubator. Untransduced T cells from the same donor as CD123 CAR-T, named C0, were used as a negative control in all experiments. On day 6, the activation beads were removed. The CD123 CAR-T cells were monitored using flow cytometry (FC) by staining the samples with an anti-CD19 antibody.

### 2.5. CAR-T Cell Generation from Thawed UCB

UCB units were removed from liquid nitrogen storage and immediately thawed in a 37 °C water bath according to the manufacturer’s instructions (recommendations for Cellular and Tissue Engineering Activities, Besançon, France). A solution of hydroxyethyl starch and sodium chloride (Voluven, Fresenius Kabi, France) was added to ¾ of the volume of cord blood, then it was washed with phosphate-buffered saline (PBS). Cells were resuspended in the RPMI 1640, supplemented with 20% heat-inactivated human serum. After a one-hour rest, the UCBMCs were isolated using Ficoll-Hypaque. The CAR-T generation and characterization followed the same process as cells from fresh UCB cells.

### 2.6. Flow Cytometry Analysis

All antibodies used to characterize the cell surface phenotype by FC are presented in Supplementary Table S1. FC analysis was performed using a BD FACS CANTO II and with FACS Fortessa flow cytometers, and the samples were analyzed using Diva software (BD Biosciences). Appropriately matched isotype controls were included for analysis. Briefly, T cells were stained for CD3, CD8, and CD19. CAR-T cells were examined for expression of CD3^+^/CD19^+^. The T cells’ differentiation profiles were determined by using commonly the used subsets T_N_ (CD45RA^+^, CCR7^+^), T_SCM_ (CD45RA^+^, CCR7^+^, and CD95^+^), T_CM_ (CD45RO^+^, CCR7^+^, and CD95^+^), T_EM_ (CD45RO^+^, CD95^+^), and T_EMRA_ (CD45RA^+^, CD95^+^).

### 2.7. CAR-T Expression

The expression of the anti-CD123 scFv on the cell surface of CAR-T was evaluated by FC. The transduction rate was determined using anti-CD3-BV421 and anti-CD19-APC (CAR-T cells are CD3+ and CD19+). To evaluate CAR expression, T cells (100,000 cells) were stained with 0.25 µg of biotinylated CD123 protein for 1 h at room temperature, followed by PE-conjugated streptavidin (BD Biosciences, San Jose, CA, USA). We evaluated the fixation of the protein CD123 on the CD3^+^ CD19^+^ CAR-T in comparison with C0.

### 2.8. In Vitro Functionality Assessment

To determine the capacity of secretion in IFN-γ and IL-2 upon target cell contact, CAR-T cells were co-cultured with the target CD123^+^CAL-1 cells at an E:T ratio of 1:1 for 15 h at 37 °C. Intracellular cytokine staining was performed with anti-human IFN-γ-BV421 and IL-2-PE using the BD Cytofix/Cytoperm™ Plus Fixation/Permeabilization Solution Kit with BD GolgiPlug™ (BD Biosciences), in accordance with the manufacturer’s information. The cytotoxicity of UCB and PB CAR-T, or C0 T cells, was evaluated after labeling by using a fixable viability Dye eFluor solution, in accordance with the manufacturer’s protocol (Invitrogen), and the cells were co-cultured with the target CD123^+^ CAL-1 cells at the indicated E:T ratio of 1:1, or 5:1, for 24 h at 37 °C for each experiment. Cytotoxicity was evaluated as previously described [35]. Targeted cell death was evaluated using 7-AAD labeling.

### 2.9. In Vivo Study

NOD/SCID/IL2Rγc-deficient (NSG) mice (6–8 weeks of age) were irradiated (2.5 Gy) and inoculated intravenously with 1 × 10^6^ of luciferase expressing CAL-1 cells on day 0. T cells from fresh UCB (UCB C0 or UCB CAR-T) were injected into the tail vein on day 3. Leukemic progression was monitored every 10 days using bioluminescent in vivo imaging (IVIS Lumina Series III; Perkin Elmer, Waltham, MA, USA) after a luciferin (XenoLight D-Luciferin, Perkin Elmer, Waltham, MA, USA) intraperitoneal injection.

In this model, mouse blood was analyzed regularly to detect the presence of human T cells (C0 and CAR123-T) (hCD3-FITC, CD19-APC, CD123-PC7) and to study the differentiation profile of UCB CAR-T by FC (hCD45-FITC, CD3-APC-H7, CD19-APC, CD8-BV510, CD45RA-V450, CD45RO-PC5.5, CCR7-FITC, CD95-PE). Survival rates were followed daily. All procedures were carried out in accordance with the guidelines for animal experimentation, according to an approved protocol (Veterinary Services for Animal Health and Protection, issued by the Ministry for Agriculture, Paris, France).

### 2.10. Statistical Analysis

Statistical analyses were performed using R 4.0.2. Visualization was performed using the ggplot2 (3.3.2), tibble (3.0.4), stats (4.0.2), ggpubr (0.4.0), and survival (3.2–7) packages. Student’s T-test was used to analyze the differences between conditions. In vitro data are presented as mean ± standard deviation (SD). Kaplan–Meier curves were compared using the log-rank test to analyze overall survival (OS). *p*-values < 0.05 were considered to be statistically significant (* *p* < 0.05, ** *p* < 0.01, *** *p* < 0.001, **** *p* < 0.0001).

## 3. Results

### 3.1. CD123 CAR-T Cells Can Be Produced from UCB T Cells

**Figure 1:**
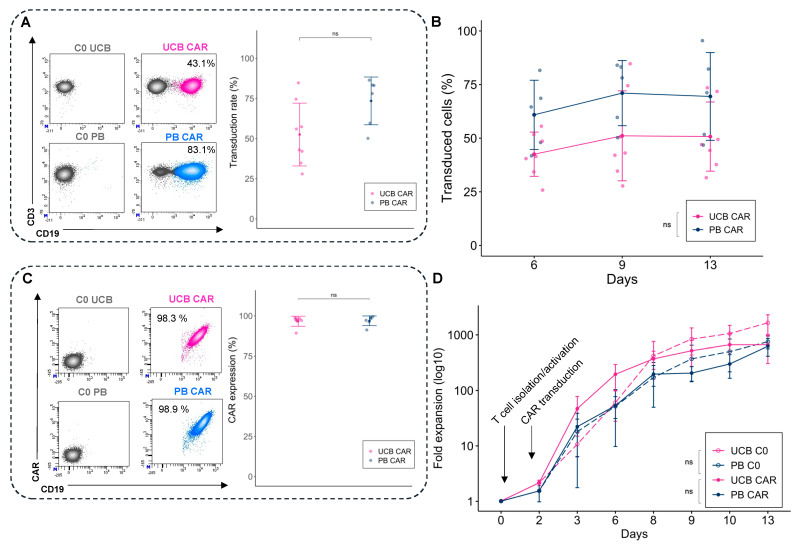
Generation of CAR-T derived from fresh UCB and PB. (**A**) Representative example of flow cytometry plots to determine the transduction efficiency (left) and mean CAR CD123 transduction rate (day 9) between UCB CAR-T (pink) and PB CAR-T (blue). (**B**) Percentage of transduced cells over time between UCB CAR-T (pink) and PB CAR-T (blue). (**C**) CAR expression using CD19: examples of flow cytometry plots (left) and percentage of CAR expression among T cells at day 9 (right). (**D**) Fold expansion of the untransduced T cells from UCB and PB (UCB C0 and PB C0), and CAR-T from UCB and PB (UCB CAR-T and PB CAR-T), after T cell isolation, activation, and transduction (*n* = 8 for UCB, *n* = 7 for PB). UCB: Umbilical Cord Blood, PB: Peripheral Blood. ns: non-significant.

Two days after activation, T cells from fresh UCB and PB were cultured with the lentiviral vector encoding the CAR123. To determine transgene efficiency, we compared the transduction efficiency (percentage of T cells expressing CD3^+^/CD19^+^) with C0. The transduction efficiency for UCB CAR-T did not differ significantly from the PB counterpart, but it tended to have lower transduction rates: 52.5 ± 19.6% for UCB CAR-T and 73.5 ± 14.9% for PB-CAR-T (*p* = 0.06) (Figure 1A). Transduction was stable for UCB and PB CAR-T until day 13 (Figure 1B). The membrane CAR expression was determined using a CD123 biotinylated protein, and it did not differ significantly between UCB and PB (Figure 1C). After transduction, both UCB T cells and PB T cells were expanded for 11 days. The fold expansion was similar for UCB CAR-T and UCB C0, and PB CAR-T and PB C0 (Figure 1D).

Overall, we demonstrated the feasibility of producing CAR-T from fresh UCB with similar proliferative capacities, transduction rates, and stability of expression compared with PB CAR-T.

### 3.2. UCB CAR-T Cells Retain a Less Differentiated Phenotype

**Figure 2:**
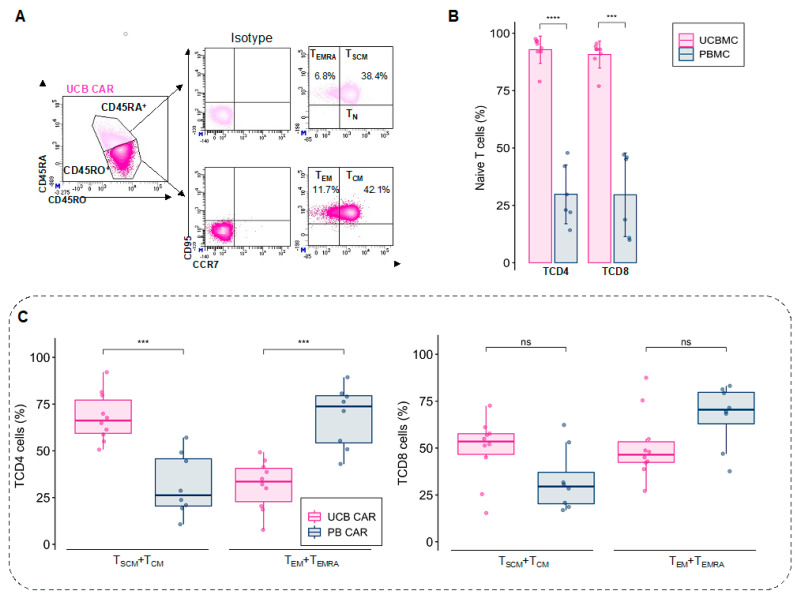
Profile of UCB-CAR-T differentiation. (**A**) Gating strategy to determine the T cell phenotype using different markers (CD45RA, CD45RO, CCR7, and CD95). Percentages of the different populations are represented among the CD3^+^ cells (parental gate). (**B**) Percentage of naïve T cells derived from fresh UCB (pink) and PB (blue) for TCD4 and TCD8 phenotypes before activation and expansion (*n* = 10). (**C**) The final product of CAR-expressing T cells (CD19+) mainly showed mainly less differentiated T cells (T_SCM_ and T_CM_) for TCD4 UCB after expansion (day 9), compared with PB CAR-T, which mainly had more differentiated T cells (T_EM_ and T_EMRA_). The difference in T cell phenotype was the same for TCD8, but it was not significantly different between UCB CAR-T and PB CAR-T (*n* = 10 for UCB and *n* = 8 for PB). UCB: Umbilical Cord Blood, PB: Peripheral Blood. ns: non-significant, *** *p* < 0.001, **** *p* < 0.0001.

Different memory T cell subpopulations (T_N_, T_SCM_, T_CM_, T_EM_, T_EMRA_) were determined in living CD4 and CD8 T cells (Figure 2A). Before activation and expansion, T cells from UCB contained a large pool of naïve T cells for TCD4 (92.8 ± 5.9%) and TCD8 (90.7 ± 5.9%), compared with PB (28.03 ± 14.5% for TCD4 and 28 ± 19.7% for TCD8) (Figure 2B and Supplementary Figure S1A).

After nine days of expansion with IL-7 and IL-15, UCB CAR CD4 T cells retained a less differentiated phenotype than its PB CAR-T counterparts, with a higher proportion of T_SCM_ and T_CM_ (68.1 ± 12.9% for UCB CAR-T and 31.8 ± 16.5% for PB CAR-T, *p* < 0.001), and a lower proportion of T_EM_ and T_EMRA_ (31.8 ± 12.9% for UCB CAR-T and 68.2 ± 16.5% for PB CAR-T, *p* < 0.001). In contrast, UCB CD8 T cells and PB showed no significant differences in their differentiation profiles, but UCB CAR-T tended towards a less differentiated profile (49.2 ± 16.9% of T_SCM_ + T_CM_ and 51.0 ± 17.8% of T_EM_ + T_EMRA_ for UCB CAR-T and 32.8 ± 16.5% of T_SCM_ + T_CM_ and 67.1 ± 16.5% of T_EM_ + T_EMRA_ for PB CAR-T, *p* = 0.055 for T_SCM_ + T_CM_ and *p* = 0.064 for T_EM_ + T_EMRA_) (Figure 2C). For the UCB T cells, transduction does not change the phenotype, in comparison with C0. (Supplementary Figure S1B).

These results demonstrate that fresh UCB CAR-T cells retain a less differentiated phenotype than PB CAR-T cells after expansion using IL-7 and IL-15, especially for TCD4.

### 3.3. UCB and PB CAR-T Cells Exhibit Similar In Vitro Functionality

**Figure 3:**
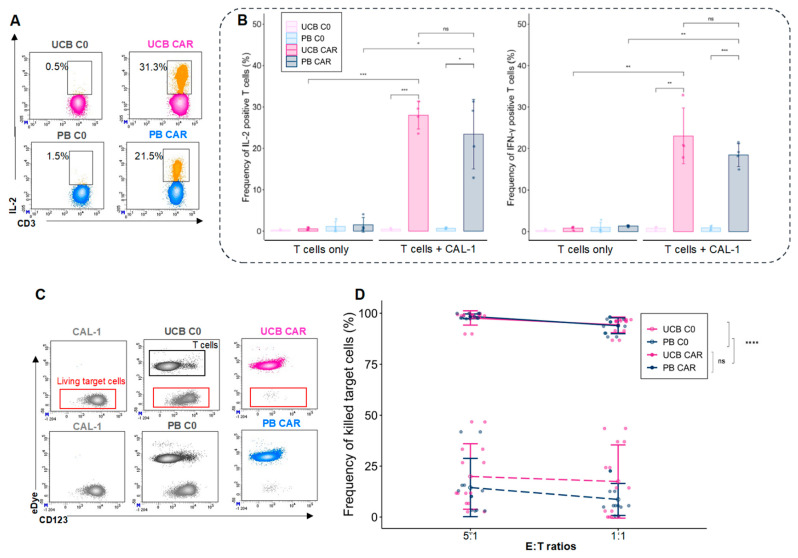
In vitro functionality of fresh UCB CAR-T. (**A**) Examples of intracellular staining of IL-2 in CD3+T cells. (**B**) Frequency of intracellular IL-2 and IFN-γ positive cells after 15 h of co-culturing T cells with CAL-1 cells at an E:T of 1:1 (*n* = 4). UCB (pink), PB (blue). (**C**) Cytotoxicity of CAR-T cells. Examples of a representative experiment to determine the cytotoxicity of target cells (CAL-1). Living target cells were defined as CD123+/Edye-/7AAD- (red boxes). eDye positive T cells are in black boxes. CAL-1: the CAL-1 line alone. UCB C0: CAL-1 plus UCB-C0. UCB CAR-T: CAL-1 plus UCB-CAR-T. (**D**) Levels of cytotoxicity after 24 h of co-culturing (5:1 and 1:1 E:T ratio) (*n* = 8 for UCB and *n* = 7 for PB). UCB: Umbilical Cord Blood, PB: Peripheral Blood. ns: non-significant, * *p* < 0.05, ** *p* < 0.01, *** *p* < 0.001, **** *p* < 0.0001.

To assess the in vitro functionality of the UCB CAR-T, we evaluated their capacity to secrete Th1 cytokines, such as IL-2 and IFN-γ (Figure 3A), and to lyse the BPDCN CAL-1 cell line during co-culture experiments. We observed no difference in terms of the intracellular expression of IL-2 (28.0 ± 3.3% for UCB CAR-T and 23.4 ± 8.4% for PB CAR-T, *p* = 0.37) nor IFN-γ (23 ± 6.7% for UCB CAR-T and 18.4 ± 2.7% for PB CAR-T, *p* = 0.27), which is in accordance with the source of the T cells (Figure 3B). UCB and PB CAR-T exhibited a strong leukemic cell-killing capacity (Figure 3C). At an E:T ratio of 1:1, we observed high cytotoxicity potentials in UCB CAR-T (94.3 ± 3.8%) and PB CAR-T (93.8 ± 3.9%), compared with UCB C0 (17.4 ± 17.9%, *p* < 0.0001 vs. UCB CAR-T) and PB C0 (8.6 ± 7.9%, *p* < 0.0001 vs. PB CAR-T), with no significant difference between UCB and PB CAR-T (*p* = 0.84) (Figure 3D). In conclusion, fresh UCB CAR-T cells possess a high lytic capacity, similar to PB CAR-T, and they can mount a cytokine response toward tumor cells in vitro.

### 3.4. UCB CAR-T Cells Exhibit Significant Antitumor Activity In Vivo

**Figure 4:**
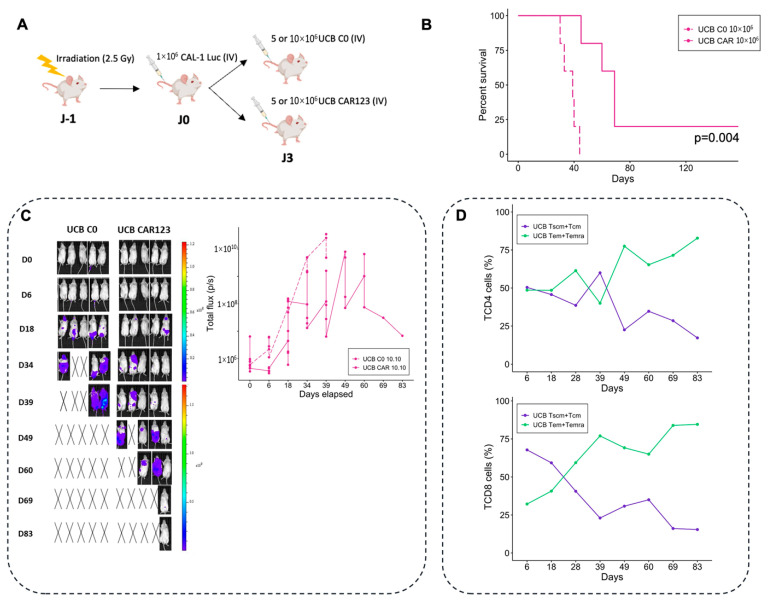
In vivo functionality of UCB CAR-T. (**A**) The experimental protocols. (**B**) Overall survival of mice treated with fresh UCB CAR-T, and the control group (C0), over time (*n* = 5 mice per group, dose: 10 × 10^6^ T cells). (**C**) Bioluminescent intensity of the mice treated with UCB CAR-T and the control groups (*n* = 5 mice per group). Black cross indicates dead mice. (**D**) Percentage of different T cell subsets (T_SCM_ + T_CM_ and T_EM_ + T_EMRA_) for UCB CAR-T (TCD4 and TCD8), for each group, over time. UCB: Umbilical Cord Blood.

To assess the in vivo antitumor activity of UCB CAR-T cells, we injected luciferase-expressing CAL-1 cells (1 × 10^6^)—and three days later, 5 × 10^6^ (low dose) or 10 × 10^6^ (high dose) T cells from fresh UCB (UCB-C0 or UCB-CAR-T)—into previously irradiated NSG mice (Figure 4A). Luminescence reflecting leukemia infiltration was higher in mice treated with UCB C0 compared with UCB CAR-T, thus demonstrating their ability to control BPDCN growth in vivo. UCB CAR-T treatment further increased the OS of mice, compared with C0 treated mice that were given a high dose (10 × 10^6^) (125 days versus 44 days, five mice/group, *p* = 0.004) (Figure 4B) and low dose of CAR-T (65 days versus 41 days, five mice/group, *p* = 0.004) (Supplementary Figure S2A). These results were confirmed in another independent experiment, with a significant improvement in terms of OS with UCB-CAR-T treated mice (*p* = 0.01, Supplementary Figure S2B). Moreover, the dissemination of leukemia cells in mice treated with CAR-T is delayed compared to mice treated with C0 (Figure 4C). Regarding the phenotype of CAR-T, we observed an increase in more differentiated T cells (T_EM_ + T_EMRA_ at day 83: 82.8% for TCD4 and 84.6% for TCD8), and the maintenance of a pool of poorly differentiated T cells (T_SCM_ + T_CM_ at day 83: 17.2% for TCD4 and 15.4% for TCD8) (Figure 4D). On day 83, the presence of UCB CAR-T in blood remained present (3052 T cells/mL, with 76.5% of CD19 expression, *n* = 1).

Thus, UCB CAR-T cells are able to control BPDCN in a dose-dependent manner, since a dose of 10 × 10^6^ of CAR-T induced better survival rates than the 5 × 10^6^ doses (*p* < 0.05, Supplementary Figure S2B). In conclusion, UCB CD123 CAR-T can differentiate into more differentiated T cells in vivo, and are cytotoxic against tumor cells.

### 3.5. Cryopreserved UCB Units Can Be Used to Produce Functional CAR-T

**Figure 5:**
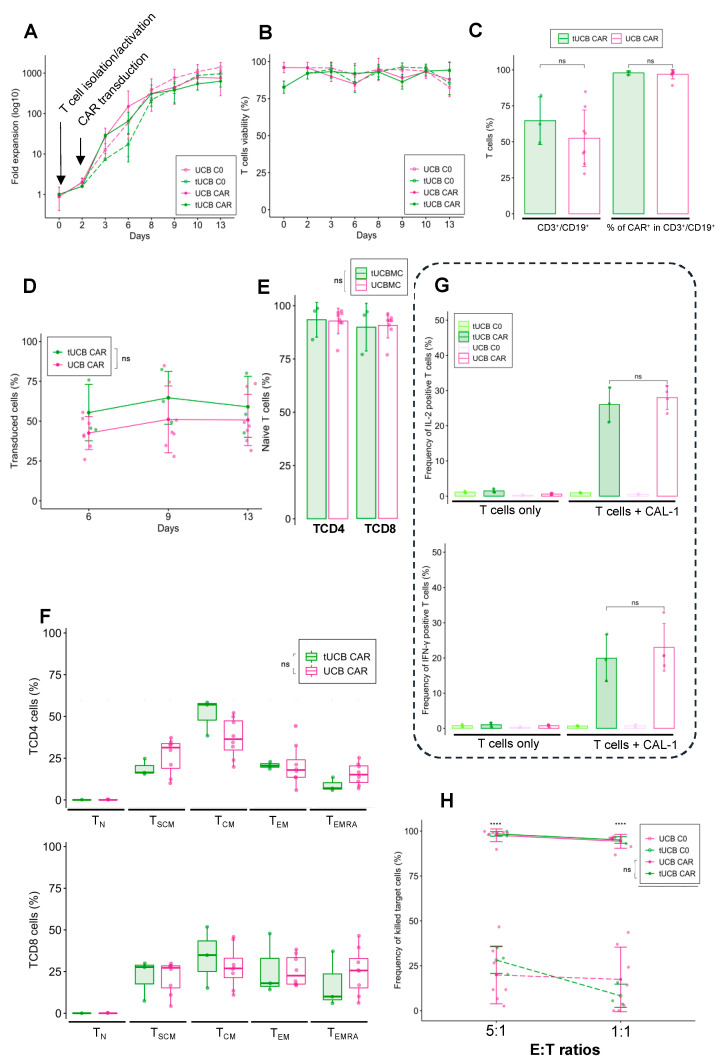
Generation of functional UCB CAR-T from cryopreserved/thawed UCB. (**A**) Fold expansion of untransduced T cells from thawed (tUCB C0) and fresh UCB (UCB C0), and CAR-T cells from thawed (tUCB CAR-T) and fresh UCB (UCB CAR-T) after T cell isolation, activation, and transduction (*n* = 3 for tUCB, *n* = 8 for UCB). (**B**) T cell viability over time, as determined using the trypan blue exclusion test, for tUCB C0, UCB C0, tUCB CAR-T, and UCB CAR-T after T cell isolation, activation, and transduction (*n* = 3 for tUCB, *n* = 8 for UCB). (**C**) Percentage of transduced cells determined by transduction rate, and CAR expression for thawed UCB CAR-T and fresh UCB (*n* = 3 for tUCB and *n* = 8 for UCB). (**D**) Transduction rate over time for tUCB CAR-T and UCB CAR-T (*n* = 3 for tUCB and *n* = 8 for UCB). (**E**) Percentage of naïve T cells derived from UCB, for the TCD4 and TCD8 phenotypes before activation (*n* = 3 for tUCBMC and *n* = 10 for UCBMC). (**F**) Differentiation profile for tUCB CAR-T and UBC CAR-T, nine days after expansion (*n* = 3 for tUCB CAR-T and *n* = 8 for UCB CAR-T). (**G**) Intracellular staining of IL-2 and IFN-γ was performed after 15 h of co-culturing T cells (CAR123 or C0), with CAL-1 cells having an E:T of 1:1 (*n* = 3 for tUCB and *n* = 4 for UCB). (**H**) Twenty-four hours after co-culturing CAL-1 cells in a 5:1 and 1:1 E:T ratio (*n* = 3 for tUCB and *n* = 8 for UCB). UCB: Umbilical Cord Blood, PB: Peripheral Blood, ns: non-significant, **** *p* < 0.0001.

In real life, UCB CAR-T could be made from cryopreserved UCB, in contrast to the production of CAR-T from PB, which uses fresh blood. In order to show that this cryopreservation phase does not alter the functionality of CAR-T, we generated UCB CAR-T from three cryopreserved UCB units. After thawing the units, the thawed UCB CAR-T (tUCB CAR-T) and thawed C0 (tC0) showed comparable proliferation and viability compared with fresh UCB cells (Figure 5A,B). Furthermore, the transduction capacity was also similar, with a transduction efficiency of 64.6 ± 16.5% for tUCB CAR-T (52.5 ± 19.6 for fresh UCB CAR-T, *p* = 0.36) (Figure 5C), and it was stable over time (after 13 days of expansion 59.0 ± 19.2% for thawed UCB and 50.7 ± 16.1 for fresh UCB CAR-T, *p* = 0.71) (Figure 5D).

We did not observe any difference in terms of differentiation profile between thawed and fresh UCB T cells before activation (93.4 ± 8.1% of CD4 T_N_, *p* = 0.92 with UCB CAR-T, and 89.9 ± 11.2% of CD8 T_N_, *p* = 0.91 with UCB CAR-T) (Figure 5E). After expansion, the phenotype remained poorly differentiated and similar to fresh UCB CAR-T, with a majority of T_SCM_ (18.9 ± 5.0% for TCD4 *p* = 0.15 with UCB CAR-T, and 21.7 ± 12.4% for TCD8, *p* = 0.99 with UCB CAR-T) and T_CM_ (51.3 ± 11.1% for TCD4, *p* = 0.14 with UCB CAR-T, and 34.0 ± 18.3% for TCD8, *p* = 0.5 with UCB CAR-T) and a minority of T_EM_ (20.7 ± 2.1% for TCD4, *p* = 0.97 with UCB CAR-T, and 26.7 ± 18.4% for TCD8, *p* = 0.91 with UCB CAR-T) and T_EMRA_ (8.9 ± 4.1% for TCD4, *p* = 0.094 with UCB CAR-T, and 17.8 ± 16.9% for TCD8, *p* = 0.55 with UCB CAR-T) (Figure 5F). Cryopreserved UCB CAR-T cells were able to recognize and target tumor cells, and they had the capacity to secrete IL-2 (26.0 ± 4.9%) and IFN-γ (19.9 ± 6.7%) in a similar manner to fresh UCB CAR-T (*p* = 0.63 for IL-2 and IFN-γ expression between fresh and tUCB CAR-T) (Figure 5G). In addition, they showed strong in vitro cytotoxicity potential against CAL-1 cells (E:T ratio 1:1, 95.03 ± 1.82%) which is identical to that of fresh UCB CAR-T (*p* = 0.69) (Figure 5H). Overall, the cryopreservation of UCB does not cause significant changes to the transduction rates, stability of CAR expression, phenotype, or functionality of UCB CAR-T.

## 4. Discussion

In recent years, the success of CAR-T therapies in treating patients with hematological malignancies by targeting CD19 or BCMA has revolutionized cancer treatment, and many CAR-T cells have been developed for use against other leukemic antigens or solid tumors [1,2,36]; however, relapses occur after CAR-T cell therapies, and several clinical studies have shown that the injection of CAR-T cells with a less differentiated phenotype, such as T_SCM_ and T_CM_, are particularly attractive because of their ability to induce long-lasting anti-tumor immunity [8,28,29,30,37]. The adoptive cell transfer of even a very low number of T_SCM_ and T_CM_ can reconstitute the maintenance of a robust and long-term immune response [38,39]. Indeed, the optimization of the CAR-T differentiation profile during production could allow for better persistence and longer immune responses in vivo. Arcangeli et al. observed that the proportion of T_SCM_ in the final CAR-T cell product positively correlated with in vivo expansion [40]. The choice of cytokines for CAR-T cells’ ex vivo expansion is also a key factor. IL-2 is frequently used for T cell expansion, but it yields the intense proliferation and differentiation of T cells [22,32]. IL-2 has been gradually replaced by other cytokines, such as IL-7, 15, 12, or 21 [21,37]. IL-7 was shown to be critical for T cell homeostasis and expansion, whereas IL-15 supported superior T_SCM_ and T_CM_ phenotype preservation compared with IL-2 [33,34,41,42]. In this study, we used IL-7 and IL-15 for PB and UCB CAR-T production, with in-house optimized concentration [43], and we managed to retain the less differentiated T cells compared with the CAR-T productions that used IL-2 (not shown).

In this context, UCB has many pragmatic advantages: UCB contains a high pool of naïve T cells (85–95%) and less differentiated T cells (T_SCM_ and T_CM_). UCB units are HLA-typed, thus making them highly qualified in terms of viability and the number of nucleated cells and cryopreserved in UCB banks. Moreover, only 5–6% of the global stock has currently been released [12,44], meaning that these banks are an ideal source for CAR-T production in the future.

Serrano et al. first reported that naïve UCB T cells could be differentiated into CD19-specific cytolytic effectors (first generation of CAR-T) [45]. Since then, only a few studies have also focused on the production of CAR-T derived from UCB [46,47,48,49,50,51,52,53]. In our work, we demonstrated the feasibility of using T cells from UCB, and we genetically modified them ex vivo to target the CD123 antigen. The culture parameters (IL-7 + IL-15) allow the preservation of the less differentiated phenotype by starting from a pool of naïve T cells. After an efficient expansion, the UCB CD4 T cells were significantly less differentiated than PB CD4 T cells, and CD8 experienced the same tendency (but that is not significant for our experiment). These results are similar to those reported by Olbrich et al. who produced UCB and PB CAR-T that redirected against cytomegalovirus in the presence of IL-7 and IL-15, thus showing that UCB TCD8 cells were more differentiated than UCB TCD4 cells [53]. This is also in line with the findings of Pegram et al. which showed that the expansion of CAR-T derived from UCB, using IL-7 and IL-15, preserved a poorly differentiated phenotype while maintaining the expansion of T cells [49]. In a previous study, different doses of IL-7 and IL-15 were tested for transgenic T cell production from UCB, and we showed that IL-7 and IL-15 doses of 20 and 10 IU/mL, respectively, were the most appropriate [43]. The specificity of CD4 T cells could explain the extraordinary persistence of anti-CD19 CD4 CAR-T cells, which can last up to 10 years, as recently described by June et al. [54].

We used a lentiviral vector for the transduction of vector encoding CAR. We obtained the variable transduction rates between units (from 27.8% to 84.8%), but the rates became stable over time. This variability has also been described in studies with UCB CAR-T, wherein the transduction efficiency varied from 15–70% [47]. This lower transduction rate could be due to the T cell differentiation profile. Indeed, Frumento et al. showed that T_CM_ and T_EM_ were the subsets with the highest transduction rates [55]. Despite this, the level of transduction efficiency obtained with our vector is relatively high (52.5 ± 19.6%), and it is not statistically different from those of PB CAR-T (73.5 ± 14.9%, *p* = 0.06). Moreover, CD123 UCB CAR-T cells present a sustained efficiency in vitro. Contrary to other published studies [46,47,49], we obtained profound leukemic cell-killing abilities at lower E:T ratios (more than 90% at a 1:1 ratio), which is similar to PB-derived CAR-T, although there are fewer effector T cells in UCB-CAR-T production. We can hypothesize that naïve T cells from UCB are able to differentiate, in vivo, into more differentiated subsets (T_SCM_, T_CM_, or T_EM_). In fact, upon tumor cell recognition, the naïve pool of T cells decreases in order to mirror the increase in more differentiated T cells.

Few studies have evaluated the functionality of UCB-derived CAR-T cells in vivo. Pinz et al. evaluated a CD4-targeted CAR-T in ALL (Acute Lymphoblastic Leukemia) models, and they showed a better control with high doses of CAR-T (minimum 10 × 10^6^ CAR-T per mouse) [52]. In our BPDCN models, we showed that UCB CAR-T significantly improves mouse survival; however, in order to evaluate UCB CAR-T persistence, more complex mouse models are required, such as the humanized IL-7 mouse model recently described by Coppin et al. [56]. Another possibility is the injection of human cytokines. For example, IL-15 is crucial for the generation and maintenance of memory CD8 T cells, and it enhances the in vivo antitumor activity [57,58,59]. Indeed, as NSG mice are highly immunocompromised, modeling the human environment seems to be necessary to activate UCB CAR-T.

It is probable that CAR-T cell therapies from healthy donors will move to allogeneic CAR-T “off-the-shelf” products in the future. UCB presents many advantages for that purpose, and ongoing clinical trials will provide evidence for their potential for antitumor cell therapy, in combination with UCB transplantation [60] (NCT01362452), or in an allogenic context [61]. The main disadvantage of this approach is GVHD and graft rejection; however, compared with PB-derived T cells, UCB T cells seem to induce less GVHD [16,62] that allows the transfer of allogeneic T cells into a donor, with only 4/6 matched HLA.

UCB units are cryopreserved in liquid nitrogen at −196 °C [63]. It was therefore necessary to ensure that the cryopreserved UCB T cells could enable the production of functional CAR-T. To the best of our knowledge, no study has evaluated the impact of UCB cryopreservation on the functionality of T cells, given that they are genetically modified after the thawing step. Micklethwaite et al. produced UCB CAR-T targeting CD19 from cryopreserved units, but they did not confront their result with a fresh UCB CAR-T comparator [48]. In our study, we observed no significant differences in terms of T cell viability, expansion rate, transduction efficiency and stability, differentiation profile, and functionality capacities, thus underlining that the cryopreservation/thawing process does not alter the functionality of UCB-derived T cells.

## 5. Conclusions

In conclusion, we generated UCB CAR-T cells that retain a less differentiated phenotype than PB-derived CAR-T cells, while maintaining a similar high level of in vitro cytotoxicity to PB CAR-T, and significant efficacy in vivo; however, in order to pave the way for the development of allogeneic CAR-T from UCB, and in order for it to be available in banks and as usable “off-the-shelf” products, these results must be confirmed in appropriate in vivo models and compared with data obtained from PB to define the place of UCB-derived CAR-T cells in future biotherapies.

## Data Availability

Not applicable.

## Supplementary Materials

The following supporting information can be downloaded at: https://www.mdpi.com/article/10.3390/cancers14133168/s1, Figure S1: Differentiation profile at day 0 and nine days after expansion; Figure S2: In vivo functionality of UCB CAR-T cells; Table S1: Monoclonal antibodies used to characterize cells by flow cytometry.

## Abbreviations

UCB	Umbilical Cord Blood
PB	Peripheral Blood
CAR-T	Chimeric Antigen Receptor T cells
BPDCN	Blastic Plasmacytoïd Dendritic Cell Neoplasm
