# Supplementary material for: Umbilical Cord Blood as a Source of Less Differentiated T Cells to Produce CD123 CAR-T Cells
Source: Cancers (Basel). 2022 Jun 28;14(13):3168. doi: 10.3390/cancers14133168 (PMC9264759; doi:10.3390/cancers14133168)
Supplement: Supplementary file 1 [file cancers-14-03168-s001.zip › SUPPLEMENTARY DATA.pdf]

**SUPPLEMENTARY DATA**

Supplemental materials and methods

**Flow cytometry**

*Table SI: Monoclonal antibodies used to characterize cells by flow cytometry.*

| Fluorochrome | Target | Provider |
|---|---|---|
| APC | **CD19** | Miltenyi Biotec |
| BV421 | **CD3** | BD Pharmingen |
| BV510 | **CD8** | BD Pharmingen |
| PE-CY7 | **CD45RA** | BD Pharmingen |
| PERCP CY5.5 | **CD45RO** | BD Pharmingen |
| FITC | **CCR7** | R&D System |
| PE | **CD95** | BD Pharmingen |
| PE | **Streptavidin** | BD Pharmingen |
| PC7 | **CD123** | Sony Biotechnologies |
| - | **7-AAD** | Sony Biotechnologies |
| FITC | **CD3** | BD Pharmingen |
| PE-CY7 | **CD8** | BD Pharmingen |
| BV421 | **IFN-γ** | BD Horizon |
| PE | **IL-2** | BD Pharmingen |

Supplemental figures

**Supplementary Figure S1: Differentiation profile at day 0 and nine days after expansion (A)** Percent of naïve T cells derived from UCB and PB for TCD4 and TCD8 phenotype before activation and expansion (n =10 for UCB and n=8 for PB). **(B)** Detailed phenotype nine days after expansion for UCB and PB CAR-T and C0 (TCD4 and TCD8) (n=10 for UCB and n=8 for PB). UCB: Umbilical Cord Blood, PB : Peripheral Blood,Ns : non significant, *p < 0.05, **p < 0.01, ***p < 0.001, ****p < 0.0001.

**Supplementary Figure S2**: ***In vivo* functionality of UCB CAR-T cells. (A)** Overall survival of mice treated with UCB CAR-T or control group (C0) over time (n=5 mice per group, dose : $5.10^6$ T cells) **(B)** Bioluminescence imaging analysis of tumor growth in NSG mice before and during UCB CAR-T or C0 treatment ($5.10^6$ and $10.10^6$ cells for each group). Black cross indicates dead mice **(C)** Overall survival of mice treated with UCB CAR-T or control group (C0) over time (n=4-5 mice per group, doses : $5.10^6$ or $10.10^6$ T cells)
