# Supplementary figures and images for: Umbilical Cord Blood as a Source of Less Differentiated T Cells to Produce CD123 CAR-T Cells
Source: Cancers (Basel). 2022 Jun 28;14(13):3168. doi: 10.3390/cancers14133168 (PMC9264759; doi:10.3390/cancers14133168)

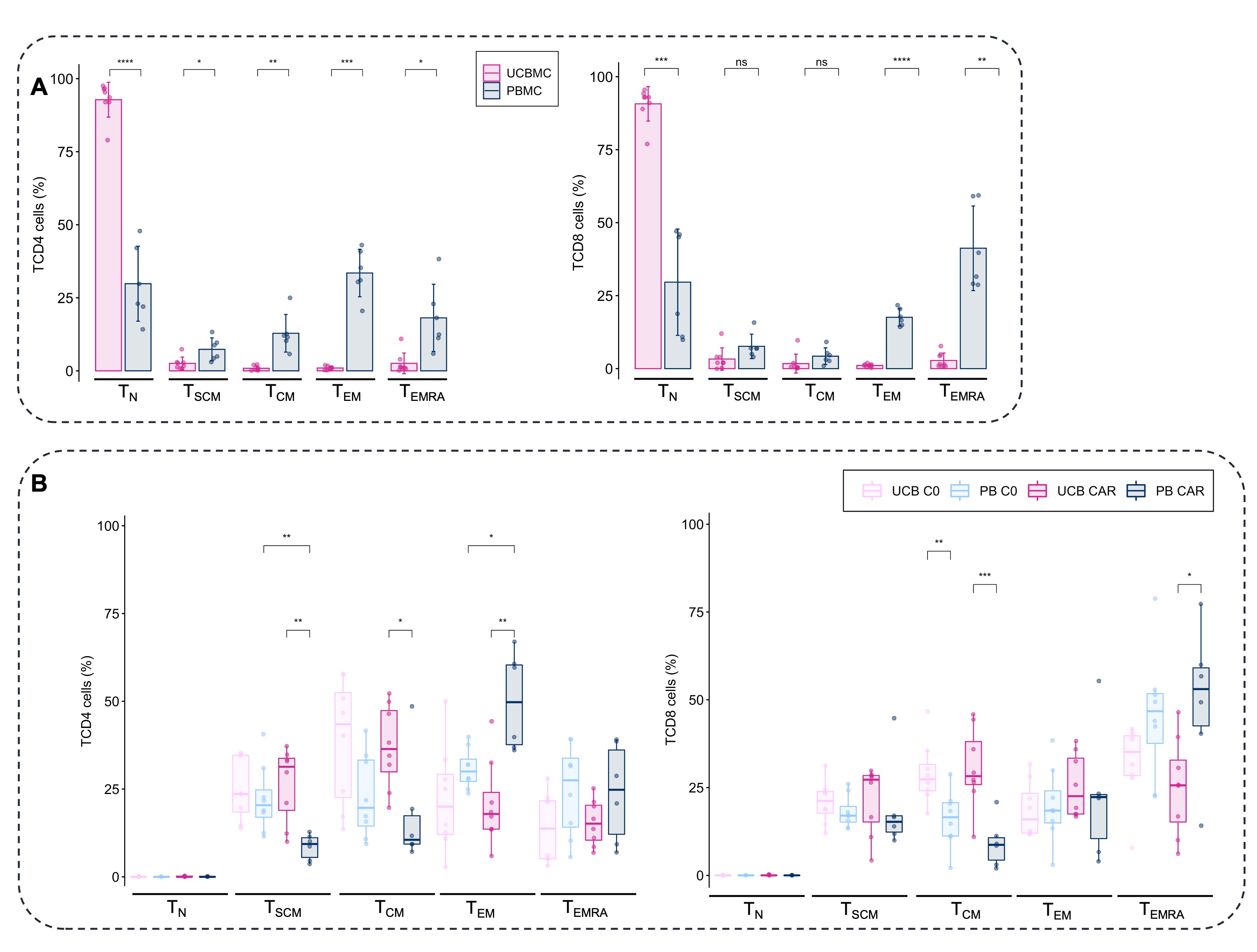

Supplement: Supplementary file 1 [file cancers-14-03168-s001.zip › Revised FigureS1.jpg]

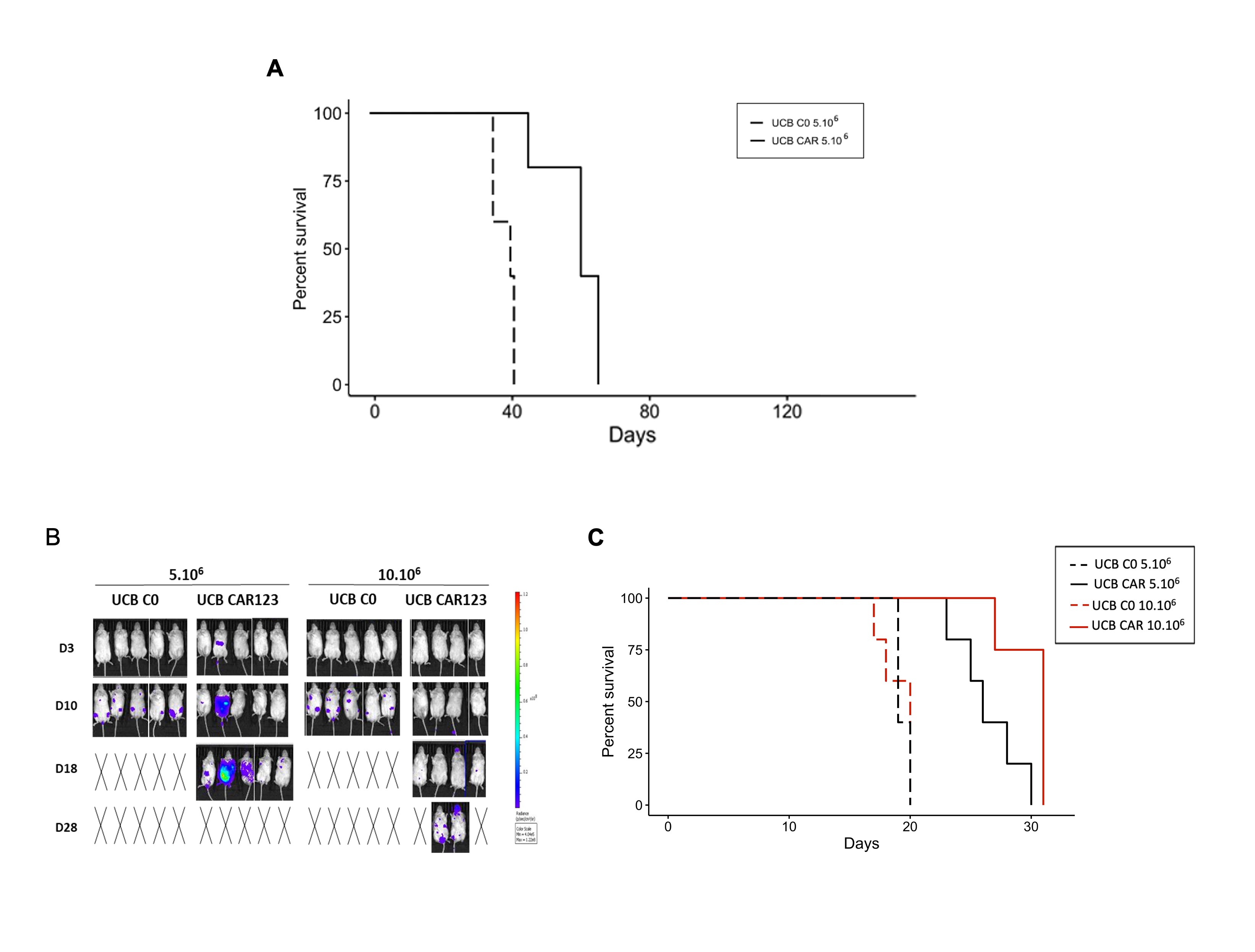

Supplement: Supplementary file 1 [file cancers-14-03168-s001.zip › FigureS2.jpg]
